# Longitudinal changes in health related quality of life in children with migrant backgrounds

**DOI:** 10.1371/journal.pone.0170891

**Published:** 2017-02-02

**Authors:** Ester Villalonga-Olives, Ichiro Kawachi, Josue Almansa, Nicole von Steinbüchel

**Affiliations:** 1 Institute of Medical Psychology and Medical Sociology, Georg-August-University Göttingen, Göttingen, Germany; 2 Department of Social and Behavioral Sciences, Harvard T.H. Chan School of Public Health, Boston, MA, United States of America; 3 Department of Health Sciences, Community and Occupational Medicine, University of Groningen, University Medical Center Groningen, Groningen, The Netherlands; TNO, NETHERLANDS

## Abstract

**Background:**

Little is known about longitudinal changes in the Health Related Quality of Life (HRQoL) among children with migrant backgrounds.

**Methods:**

The sample comprised 350 children with predominantly migrant backgrounds enrolled in 7 kindergartens in Frankfurt and Darmstadt, Germany. At baseline, the participants’ mean age was 4.4 years (SD 0.9). Data collection started in May 2009. Two waves of data were collected one year apart (94% response rate). HRQoL was evaluated with the Kiddy-KINDL. The other variables under study were sex, age, socioeconomic status, country of origin, developmental status (WET) and individual behavior (VBV). Data were collected from the children, parents and teachers. Structural equation modeling (SEM) was used to assess the Wilson and Cleary theoretical framework on changes in HRQoL and Generalized Estimated Equations (GEE) to model the longitudinal trend in HRQoL.

**Results:**

Overall HRQoL remained stable between baseline and follow-up. SEM model fit was χ2 = 8.51; df = 5; p = 0.13; SRMR = 0.02 RMSEA = 0.06 and indicated that there were differences in kindergarten activities (p<0.05). The GEE model elucidated that the differences in HRQoL between the baseline and follow-up varied according to kindergarten activities that the children were assigned to (music, art, or no activities) (p<0.05), but that there were no differences in terms of country of origin. On average, girls reported better HRQoL.

**Conclusion:**

Overall HRQoL scores remained stable over follow-up in a sample of migrant children and there were no differences in terms of origin. However, there was heterogeneity in the results depending on the kindergarten activities that the children were assigned to.

## Introduction

Health Related Quality of Life (HRQoL) is a concept that represents a person’s own judgement of his or her subjective health status, functioning, and well-being in the domains of physical, psychological, social and role performance [1]. Measures of pediatric HRQoL combine different components such as perceived health, health behavior and well-being. Instruments have been developed to assess HRQoL in both general and specific populations. HRQoL allows identification of specific groups with high rates of unrecognized conditions, social and emotional problems, and poor functioning [2]. HRQoL in children is relevant for evaluating medical outcomes of interventions and treatments [3]. HRQoL is also useful as an indicator of emotional and social aspects of living in situations such as migration or disadvantaged socioeconomic circumstances.

HRQoL can change over the life course. In contrast to rather well documented changes in HRQoL in young populations with chronic conditions (such as cancer, oral health conditions or obesity) [4–8], there is limited information concerning changes in HRQoL due to developmental factors in healthy populations. Such information is needed as a reference for evaluating nonclinical and clinical interventions in children [9]. Some studies in young populations (ranging in age from 8 to 18 years) have found that HRQoL tends to decrease during certain points in the life course (e.g. during adolescence). These changes are attributed to pubertal onset and changes in self-esteem [10,11]. Data about trajectories of HRQoL at very early ages in children with immigrant backgrounds is important since the percentage of people currently living in Europe that come from abroad is increasing dramatically [12].

Among previous studies that evaluated HRQoL in a general pediatric population focused on preschoolers (of 2- to 4- and 4-to 6-year-old) comparing children with and without migrant parents from two separate culturally diverse populations [13]. Results suggested that in a culturally diverse population, children of migrant parents report lower HRQOL scores than children of non-migrant parents. The impact on HRQoL of children of both parents being migrants was comparable to having a chronic disease. Another report from the Center of Disease and Control (CDC) analyzed changes in pediatric populations in the US between 2001 and 2010 using several cross-sections [14]. In that study, adolescents’ self-rated health was fairly stable from 2001 through 2004 but worsened afterward. Another report analyzed changes in mental health and its effect on HRQoL using a longitudinal follow-up of children and adolescents [10]. The authors found that HRQoL worsened over 3 years, with a marked decline in those whose mental health deteriorated. Another report analyzed changes in HRQoL following the transition to secondary school [15], and showed that over the first 10 weeks of secondary school there was a meaningful improvement in HRQOL and psychological satisfaction for the majority of students. However, the analysis of changes in HRQoL in general pediatric and general migrational populations remains scarce. More longitudinal research is needed in order to learn about the trajectories of perceived health in children in general populations.

In a recent publication, we validated the Wilson and Cleary model of HRQoL in a sample of kindergarten children with predominantly migrant backgrounds [16]. In our cross-sectional analyses, the most important variables that affected HRQoL were the kindergarten groups and developmental status (cognitive and sensory-motor), whereas socioeconomic status and individual behavior also played a role in HRQoL. We provide more details about the validation of the model and variables selection in the aforementioned publication [16]. In the current study, we sought to characterize changes in HRQoL of this population over time using the same theoretical framework, and to investigate factors determining the course of HRQoL. We hypothesized that age, gender, socioeconomic status, the developmental status and kindergarten groups will have an effect on the changes of HRQoL during the follow-up. In the current study, we sought to characterize changes in HRQoL of this population over time using the same theoretical framework, and we investigated factors determining the course of HRQoL. We hypothesized that age, gender, socioeconomic status, the developmental status and different kindergartens with different pedagogic activities will have an effect on the changes of HRQoL during the follow-up. We also investigated if HRQoL depends on the migrational origin of the children if it improves over time.

## Materials and methods

### Population

Children with predominantly second generation immigrant backgrounds between 3 and 5 years of age took part in a longitudinal (three time points) study investigating the development of cognitive, emotional and psychosocial outcomes, and HRQOL. Participants were divided into three groups based on pedagogic activities to observe possible differences in HRQoL at follow-up. Group 1 was assigned to no special activities, group 2 followed music activities, and group 3 was assigned to painting activities designed by the principal investigators of the study. This decision was based on the requirements of equal treatment to have all the participants located in the same kindergartens following the same activity. These activities were not randomly assigned in this study. The activities were held twice per week for 45 minutes. At baseline, the participants mean age was 4.4 years (SD 0.9). Follow-up data at the second time point was collected one year after the first assessment, a period which was considered sufficient to observe changes in HRQoL. The study was conducted in seven kindergartens in Frankfurt/Main and Darmstadt, Germany. Participants were enrolled at the kindergartens and 96% of the parents consented to take part in the study (N = 357). Among these children, 68.1% had both parents who were born and raised outside Germany and were immigrants. Data collection started in 2009 and it is still ongoing. In this study we only used longitudinal data of the first two waves. The overall response rate was 94% (N = 350).

### Procedure

The project was approved by the local ethics committee of the University Medical Center Göttingen. Written informed consent was obtained from the participating families, together with the approval of the kindergarten councils. Families received detailed information regarding the background and implementation of the study and were offered the opportunity to withdraw their children from the study at any time. Interviewers were trained by the scientists who designed the study project and had a manual to follow detailed instructions. The collection of data was performed using face-to-face interviews with children, parents and teachers, separately. Data were collected at baseline and one year after.

### Instruments

#### Health-related quality of life

The Kiddy-KINDL (KK by Ravens-Sieberer et al, 1998) is a questionnaire assessing HRQoL in children aged 4 to 7 [3,17,18]. The recall period of the questionnaire is the past week. The short version of this questionnaire includes 12 items belonging to 6 dimensions: physical and psychological well-being, self-esteem, family, friends, and everyday functioning at the kindergarten. Response categories are associated with a 3-point Likert scale (never; sometimes; very often). Scale scores of the KK range from 0 to 100, with higher scores indicating better HRQoL. The final scores are T-scores (20–80), with higher scores indicating better HRQoL. Psychologists and educators used the interview form of the KK to collect self-reports from children who were 3 to 5 years old. Psychometric properties of the instrument in this sample were previously tested and the overall reliability and validity were found to be acceptable to very good. Cronbach’s alpha was 0.75 and overall Guttman’s lambda 0.77; for the whole scale Spearman-Brown test for split half reliability resulted in 0.80 and ICC for test-retest in 0.83. Discriminant validity investigation differentiated groups regarding SES and age [19]. Table 1 contains a list of instruments with indicators and examples.

**Table 1:** Indicators used in the study to test the Wilson and Cleary theoretical framework in pediatric data: concepts, measured variables and details of the instruments used.

Concepts	Measured variables	Instrument	Recall period	Respondent	Content example
**HRQoL**	HRQoL	Kiddy-KINDL	Past week	Children	Had fun at the kindergarten
**Environmental factors**	Socioeconomic status	Specific questions	At present	Parents	Level of education, and current job
**Functional status**	Development status	WET	At present	Parents and Children	Put on the shoes, assists in housework
**Characteristics of the individual**	Individual Behavior	VBV 3–6 scale	Past four weeks	Kindergarten Teacher	Shows feelings spontaneously

#### Socioeconomic variables

Information about occupation and level of education of the main breadwinner of the family was collected during an interview with the parents in order to characterize the socioeconomic status. Here the international ISCO categorization was followed. The final categorical variable created was: unemployed (0), unskilled workers (1), skilled workers (2), professionals (3) and professionals with advanced qualifications and managers (4). Socio-demographic and socioeconomic variables were only collected at baseline.

#### Cognitive and sensory-motor tests

The Wiener Entwicklungstest (WET by U. Kastner-Koller and P. Deimann, 2002) is a widely used instrument which measures the developmental status of children aged 3 to 6. The WET consists of 13 subtests applied to the children and a parent questionnaire covering 6 functional areas of development: visual, motor, learning/memory, cognitive stage, language, and socio-emotional development. The variables range from 0 to 9, with higher scores indicating better development. Krampfen et al validated the instrument and Cronbach’s alpha revealed values between 0.66 and 0.90, spilt-half reliability lied between 0.72 and 0.91. Differential, factorial and convergent validity of the WET was empirically supported [20]. The instrument developers, Kastner-Koller et al, determined that the instrument has good face validity and good construct validity [21]. WET was administered and tested by psychologists and educators using face-to-face interviews with parents and children.

#### Behavioral rating

The Verhaltensbeurteilungsbogen für Vorschulkinder (VBV—Behavioral Assessment Rating Scale for Preschool Children. VBV 3–6 by M. Döpfner, W. Berner, T. Fleischmann et al., 1993) is an observation and rating scale for behavioral problems with 93 items organized in 4 scales: social-emotional competence, oppositional-aggressive behavior, attention deficit/hyperactivity versus playing time and emotional disorders. Responses are arranged on a 5-point rating-scale (never; once a week; several times a week; every day and several times a day). A summary score for each subscale as well as an overall score can be calculated and these can be transferred into stanine norm scores (ranging from 1 to 9) with higher scores indicating more appropriate behavior. In this study, only information about the social-emotional behavior was collected. Kindergarten teachers rated the scales based on observed behavior in the last 4 weeks. Döpfner et al validated the instrument and Cronbach’s alpha for kindergarten teachers rated scales lied between 0.86 and 0.95. Estimates of interrater-reliability were available for a representative sample. The scales revealed good retest reliability (4 weeks; rtt = 0.72 to rtt = 0.82). Thus, the psychometrical properties are acceptable to good [22].

### Statistical analysis

For all instruments, items with missing values were imputed using the mean of the remaining items—only when the amount of missing items was under 33% on any given scale. Firstly we evaluated the adaptation of the Wilson and Cleary theoretical framework to investigate changes in HRQoL and its determinants, applying structural equation modeling [23,24]. We computed change in HRQoL, developmental status and individual behavior with follow-up minus baseline values of each scale. Model fit was evaluated using χ2, and Standardized Root Mean Square Residual (SRMR) [25]. The values to accept the model should be non-significant in the χ2, and be lower than 0.07 in the SRMR. The model was fit using maximum likelihood in M-Plus 7.1 [26]. 202 children had sufficient information to be included in the analyses.

Secondly, we performed generalized estimating equations (GEE) to model the development of HRQoL over time [27]. The outcome of this model was changes in HRQoL (follow-up minus baseline values). The sociodemographic variables (gender, SES and country of origin) and kindergarten groups were introduced in the model with dummy coding, and age was introduced as a continuous variable. Only sociodemographic variables were included in this model as the other variables (developmental status and individual behavior) did not have a significant effect (p>0.05) in previous bivariate models, neither did they show an effect in preliminary GEE analyses or previous SEM models. Indeed, by doing this, individuals with missing values for developmental status and individual behavior scales can be included in the GEE analysis, thus, improving statistical power. We included interactions with time/follow-up to observe significant group differences in their changes in HRQoL. The GEE model took into account the correlations between repeated measurements within the same subject. An exchangeable correlation matrix was used, so that all off-diagonal elements of the correlation matrix were equal [28]. Additional adjustment for HRQoL baseline values was discarded to avoid the induction of bias [29–32]. In this case, considering the variables included in the analysis and taking account the missing data, 284 children had sufficient information to be included in the analysis.

## Results

Girls represented 52.3% of the participants (Table 2). In almost 10% of the families one parent was unemployed. 31.9% of the children had at least one native German parent, while the second most important group was from Asia (predominantly from Turkey; 29%), followed by Africans (mostly from Ghana and Morocco; 22%). The overall HRQoL at baseline was 69.7 (SD 17.7) and at follow-up was 70.5 (SD 15.7). In boys and girls an increment in HRQoL between baseline and follow-up was observed, which however was not statistically significant (Table 3). Children coming from families with higher socioeconomic levels (i.e. fathers were professionals with advanced qualifications and managers) (N = 50) had an increase in HRQoL over the follow-up of a year while other SES categories remained stable (p = 0.03). With regard to kindergarten activities, group 1 (no intervention) showed a notable increment in HRQoL in wave 2, while in group 2 (musical activities) a significant decrease was observed. No significant differences were noted for group 3 (painting). There was some evidence of regression towards the mean, i.e. HRQoL scores differed between some kindergartens at baseline and converged towards the mean at follow-up.

**Table 2:** Descriptive statistics of the participants.

	Baseline (Mean SD, %)
Age	4.4 (0.9)
Development status (baseline)	4.4 (1.1)
Individual behavior (baseline)	4.6 (2.3)
Development status (follow-up)	4.8 (1.1)
Individual behavior (follow-up)	5.5 (2.0)
Gender	52.3% (girls)
Socioeconomic conditions*	
Unemployed	9.8%
Unskilled workers	15.6%
Skilled workers	46.0%
Professionals	14.3%
Professionals with advanced professions and managers	14.3%
*Kindergarten groups*	
Group 1 (no activities)	40.3%
Group 2 (music)	27.4%
Group 3 (painting)	32.3%
*Origin*	
Germany**	31.9%
Asia	29.0%
Africa	22.0%
East Europe	11.6%
Others***	5.5%

Note

*The socioeconomic variables were only collected once.

**At least one parent coming from Germany.

***Others include Western Europe and America.

**Table 3:** HRQoL at baseline and follow-up by the study variables.

	Baseline (Mean SD)	Follow-up (Mean SD)	P value paired T-test
Boys	68.5 (17.6)	69.7 (15.8)	0.61
Girls	70.7 (17.8)	71.3 (15.6)	0.94
*Socioeconomic conditions*			
Unemployed	72.4 (17.2)	71.8 (17.1)	0.99
Unskilled workers	68.2 (18.7)	66.8 (16.9)	0.34
Skilled workers	71.2 (16.4)	69.5 (15.8)	0.43
Professionals	68.9 (15.7)	71.0 (14.9)	0.47
Professionals with advanced qualifications and managers	63.2 (18.9)	73.7 (14.9)	0.03
*Kindergarten groups*			
Group 1 (no activities)	65.0(17.60)	71.7 (15.30)	0.00
Group 2 (music)	76.7 (15.40)	69.8 (15.80)	0.01
Group 3 (painting)	69.2 (16.20)	70.0 (16.10)	0.97
*Origin*			
Germany	68.3 (17.3)	71.5 (15.6)	0.25
Asia	69.7 (16.8)	68.1 (17.3)	0.30
Africa	69.3 (17.7)	72.2 (15.1)	0.36
East Europe	73.2 (16.0)	70.8 (13.1)	0.93
Others	69.7 (20.6)	71.3 (16.9)	0.88

**Fig 1:**
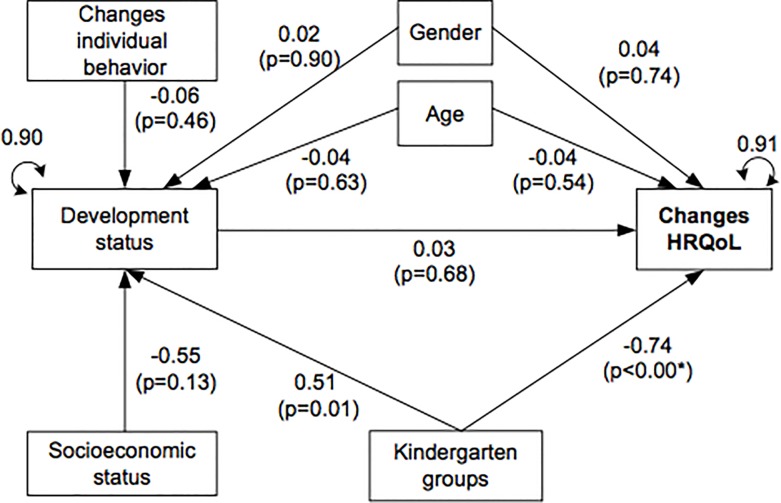
Theoretical framework to study changes on HRQoL: measurement variables and standardized estimates (β, p values and residual variances). Note: χ2 = 8.51; df = 5; p = 0.13; SRMR = 0.02 RMSEA = 0.06 Standardized coefficients are given. *P value and coefficients with the highest effect in categories of the dummy variables socioeconomic status and kindergarten groups. Ref category in gender are females.

Fig 1 shows the model factors that were hypothesized to affect HRQoL changes in children. The model fit was χ2 = 8.51; df = 5; p = 0.13; SRMR = 0.02 RMSEA = 0.06, and the overall variance of changes in HRQoL explained by the model was about 8.8%. The kindergarten activities significantly affected the changes in HRQoL with differences between groups. The group interventions had negative results compared to the group with no interventions. Changes in developmental status were positively affected by group 3 (painting). This group had over time an increase of developmental status significantly higher than the no-intervention group.

Table 4 shows the results of the GEE model. Girls had on average higher HRQoL than boys, and both genders showed similar increment at follow-up. At baseline, kindergarten groups 2 and 3 reported higher HRQoL than group 1, by 13.3 and 6.9 points respectively. We also found a significant interaction between intervention groups and the follow-up variable, supporting the notion that the change in HRQoL between baseline and follow-up was different across these groups. Group 1 showed 6.4 points of increment in the HRQoL score at follow-up; in group 2 a deterioration of 9.3 (6.4–15.7 = -9.3) points in HRQoL was observed and group 3 remained stable between baseline and follow-up (change of 6.4–7.6 = -1.2 points).

**Table 4:** Longitudinal modelling on HRQoL scores. Generalized estimating equations (GEE) adjusted for gender, age, socioeconomic and sociodemographic conditions.

	β (SE)	P value
Intercept	61.2 (4.4)	
Gender (ref. females)	-3.8 (2.0)	0.05
Age	1.8 (0.8)	0.03
*Socioeconomic conditions*		
Unemployed	Ref category	0.65
Unskilled workers	-4.1 (2.9)	
Skilled workers	-2.1 (2.6)	
Professionals	-2.0 (2.9)	
Professionals with advanced qualifications and managers	-3.5 (3.0)	
*Origin*		
Germany	Ref category	0.44
Asia	-2.9 (1.8)	
Africa	-1.3 (2.0)	
East Europe	0.5 (2.0)	
Others	-0.6 (3.5)	
*Kindergarten groups*		
Group 1 (no activities)	Ref category	<0.01
Group 2 (music)	13.3 (2.4)	
Group 3 (painting)	6.9 (2.3)	
Follow-up (ref. baseline)	6.4 (2.8)	0.03
*Interaction kindergarten group & follow-up*		<0.01
Group 2 & follow-up	-15.7 (3.7)	
Group 3 & follow-up	-7.6 (3.4)	
*Interaction gender & follow-up*		0.60
Gender (male) & follow-up	1.6 (2.9)	

## Discussion

Our results revealed that, overall, the HRQoL of our sample remained stable. However, there is an important heterogeneity in the results depending, for the most part, on the kindergarten activities.

Children in group 1 (no intervention) showed very low levels of HRQoL at baseline but by follow-up they were comparable to those in the other two groups. This significant change in HRQoL between baseline and follow-up in the intervention groups can have several explanations and mechanisms. On the one hand, this result could be explained by regression to the mean. On the other hand, the differences may be due to dissimilarities in the pedagogical activities used in the different kindergartens located in different neighborhoods. Neighborhoods with higher average education are more likely to be associated with the provision of decent housing, safe playing areas, transport, green spaces and street lighting–all of which makes the community feel good about a place and leads to greater levels of interaction and community participation, and consequently, better HRQoL. It is important to note that the kindergarten activities were not randomly assigned in this study, and hence we cannot rule out the role of chance in contributing to the differences that we observed. Emphatically, our findings should not be interpreted to mean that music activities result in worsening of HRQoL.

Second, gender played a role in our study. HRQoL was higher for girls, and changes in HQRoL were similar for both genders. A common theme in the literature is that these gender differences do not stay stable over time. Social and structured forms of play emerge systematically earlier in girls than in boys leading to subsequent differences in favor of girls at some ages [33]. As we specified in the introduction, HRQoL and developmental transitions may follow different courses for girls and boys. We observe no statistically significant differences in our results and we can’t evaluate the instability of gender differences. In our study, kindergarten children are still early in their developmental stage [14], and we did not demonstrate significant differences with increasing age. However, most children begin to experience peer social interactions at very early ages and these gender differences can start to be observed in early stages of development [33].

Third, for groups of origin, we didn’t observe differences between the groups. We observed no differentiated change in HRQoL over time. In the case of children with migrant backgrounds, it has been observed that their improvement in HRQoL may be attributed to a process of cultural adaptation and assimilation to the conditions of the country they live in now. However, the process of acculturation will depend on family characteristics (mainly socioeconomic status) and original culture [34]. Other studies yet propose discrimination towards immigrants and/or missing acculturation [35]. We didn’t observe this pattern, neither do we have data about discrimination, acculturation or social capital to interpret these results further.

Some limitations of our study deserve comment. First, our results are based on a selected kindergarten sample and results are not representative. Second, the relatively short interval between baseline and later assessment may not have been sufficient to observe meaningful changes. Third, we lacked information about assimilation, experiences of discrimination, and bridging social capital, which are likely to affect HRQoL among immigrants. Fourth, we cannot conclude that the changes we observed in HRQoL are also clinically significant. Fifth, all missing values were assumed to be completely at random. Finally, not all data correlation due to the nested structure within the kindergartens might be captured in the analyses. We decided that the additional correlation between individuals from the same kindergarten that is not explained by the intervention group would have a minimum impact on our results because of the following assumptions: 1) the strongest correlation in the data is between the repeated observations measured within the same individual, and this has been taken into account in the GEE model, and 2) the correlations within the kindergarten are largely explained by the intervention group. A multilevel analysis with the data nested in kindergartens was not considered because the number of groups was not enough to test such a model.

Our study also has several strengths. To our knowledge, this is one of the first studies that investigated the determinants of HRQoL using a theoretical framework in very young children and children with migrant backgrounds. In addition, the longitudinal design of the study provides with more appropriate basis to investigate changes in HRQoL, an important feature that has been rarely investigated in young ages and in a population with predominantly migrant backgrounds.

Our study is a contribution to the call for more tests of theoretical frameworks that elucidate the risk and protective factors of HRQoL in very young children [36]. We observed that children with migrant backgrounds tend to, on average, remain stable in terms of HRQoL with the passage of time probably due to an acculturation effect. Gender differences existed at the beginning of the study in terms of developmental status, which is in line with the literature [37]. However, gender, developmental status and origin didn’t affect the change in HRQoL. Our results suggest that the kindergarten activities, as well as the neighborhood can potentially influence children’s HRQoL. They also suggest that school-based interventions should consider the residential context of immigrants. Our results confirm the importance of longitudinal studies. Longitudinal surveys are needed to provide an in-depth look at the relative contribution and roles played by individual and contextual characteristics in HRQoL, and to study the life course of kindergarten samples.

## Supporting information

S1 File GEEDatabase. Database used for the GEE analysis.
(XLSX)

S2 File SEMDatabase. Database used for the SEM analysis.
(XLSX)
